# A retrospective analysis of dengue fever case management and frequency of co-morbidities associated with deaths

**DOI:** 10.1186/1756-0500-7-205

**Published:** 2014-04-01

**Authors:** Muhammad Arif Nadeem Saqib, Ibrar Rafique, Saira Bashir, Arsalan Ahmad Salam

**Affiliations:** 1 Pakistan Medical Research Council (PMRC), Head Office, Shahrah-e-Jamhuriat, G-5/2, Islamabad, Pakistan; 2 Pakistan Medical Research Council (PMRC) Research Centre, Fatima Jinnah Medical College, Lahore, Pakistan; 3 Pakistan Medical Research Council (PMRC) Research Centre, King Edward Medical University, Lahore, Pakistan

**Keywords:** Dengue, Co-morbid, Hypertension

## Abstract

**Background:**

Dengue epidemic in Lahore (2011) resulted in hundreds of deaths and affected thousands. As most of the studies were focused on its diagnosis and treatment, scanty data is available on associated diseases/co-morbidities in these patients that could have contributed to a higher mortality. There were no local guidelines available on recording, reporting and management of these co-morbidities. The objective of this study was to analyze the initial presentations of dengue cases and to estimate the frequency of co-morbidities in dengue patients.

**Methods:**

Data of 556 dengue cases was retrieved from 2 major public sector tertiary-care hospitals for patients who were admitted during 2011 epidemic and a case record analysis was done. Data was retrieved from patient’s information reports which included demography, signs and symptoms and the laboratory investigations. In addition verbal autopsy of deceased cases was also done from their relatives using standardized WHO verbal autopsy form after making modifications as per needed.

**Results:**

Of 556 cases studied, 390 (70%) were males. The mean age was 36 years and 30% of the cases were between 20-29 years. Average duration of the hospital stay was 6 days. Out of the total, 435 (78%) were dengue fever (DF) cases followed by dengue hemorrhagic fever (DHF) in 95 (17%) and dengue shock syndrome (DSS) in 26 (4%) cases. A total of 40 cases died and among them 17 were diagnosed with DSS, 13 DF and 10 DHF. Further the verbal autopsy from relatives of deceased cases showed 29 (60%) deceased had co-morbid diseases which included hypertension, diabetes etc. DSS was common in patients who had hypertension (27) either alone or associated with other illnesses.

**Conclusions:**

Co-morbidities with dengue infection were seen in 60% deceased cases indicating the reasons for high dengue related complications and death.

## Background

Dengue fever emerged from Africa almost 500-600 years ago and reached Asia in 1780’s [1]. In recent decades, it has become the second most prevalent mosquito (*Aedes aegypti*) borne infection after malaria [2]. Dengue has affected almost 120 countries with high incidence in many [3] putting half of the world’s population at risk [4]. According to WHO estimates, currently there are 50-100 million dengue cases around the world [5]. Dengue virus can lead to a spectrum of diseases ranging from sub-clinical infection to dengue fever and most severe forms like dengue hemorrhagic fever and dengue shock syndrome [6].

Co-morbidities in dengue patients result in complications leading to deaths. A study from Singapore reported that out of every 27 deaths due to dengue, 21 had co-morbidities [7]. Another study reported that dengue patients with allergies or diabetes are 2.5 times more at risk of developing dengue hemorrhagic fever [8]. Likewise higher frequency of complications is reported in dengue patients suffering from hepatitis [9-11].

Verbal autopsy is an indirect method of determining cause of death based on interviews with the care takers of deceased individuals and has been widely used to collect information on cause specific mortality [12]. In addition to gathering information on cause of death, verbal autopsy is also important for investigating outbreaks due to infectious diseases [13]. Many countries use verbal autopsy methods on large scale to assess cause of death in a population [14,15]. The present study was proposed to analyze the initial symptoms of dengue fever and to determine the frequency of co-morbidities in deceased cases.

## Methods

This was a retrospective, cross-sectional study and was conducted in the community of Lahore and hospital records of two tertiary care hospitals i.e. King Edward Medical University, Lahore (Mayo Hospital) and Fatima Jinnah Medical College, Lahore (Ganga Ram Hospital). Complete medical record of all dengue cases which included signs and symptoms, method of diagnosis, management, duration of stay and clinical outcome was retrieved from patient’s information reports. The diagnosis of patients into DF, DHF and DSS was taken from their case record and analyzed accordingly.

The relatives of deceased were interviewed using WHO verbal autopsy guidelines (Additional file 1). Verbal autopsy questionnaire were pre-tested by interviewing attendants of fewer cases and modified as per needs. All interviews were done by trained interviewers in local language i.e. Urdu. A written informed consent was taken from relative of deceased who were attendants of patient during the hospital stay (father, brother, sister or others). Demographic, epidemiological, co-morbidity and other details were obtained from the relatives. A sample size of 60 deceased was calculated for Lahore, however verbal autopsy of 48 deceased cases was done (40 were those whom addresses were retrieved from the records of the selected 02 hospitals while 08 were included during field interviews). These 08 cases were admitted in any other hospitals of Lahore and died. The ethical clearance was taken from Institutional ethic committee of Pakistan Medical Research Council.

### Statistical analysis

Data collected was double entered in, cleared and coded using Excel sheet (Window 2007) and analysis was done using SPSS 16.0. Chi-square test was used to compare the categorical variables and p ≤ 0.05 was considered statistically significant.

## Results

Of the total 556 cases, 390 (70%) were males and 166 (30%) females with a mean age of 36 years and majority in 20-29 years age group. Average duration of hospital stay was 6 days. The data showed that 435(78%) cases had DF, 95 (17%) had DHF and 26 (4%) DSS. Majority (64%) was positive for both IgG and IgM and pattern of positivity in DF, DHF and DSS is given in Table 1. Five day record of symptoms showed that all patients had a high grade fever (103°F) at the time of admission which settled or subsided by 5^th^ day of admission. Fever, bleeding, headache, vomiting, abdominal pain, rash and shock were common symptoms on admission. However, bleeding, vomiting, abdominal pain and rash were strongly associated with DSS (Table 2).

**Table 1:** Dengue serology of DF, DHF and DSS cases

**Test**	**DF (n 435)**	**DHF (n 95)**	**DSS (n 26)**
IgG	48	22	2
IgM	85	40	4
IgG + M	302	33	20

**Table 2:** Pattern of clinical presentation on day 1 of dengue cases

**Symptoms**	**DF (n = 435)**	**DHF (n = 95)**	**DSS (n = 26)**
Fever	435 (100%)	93 (97%)	26 (100%)
Bleeding	64 (15%)	56 (59%)	**18 (72%)
Headache	326 (75%)	24 (25%)	**24 (92%)
Vomit	323 (74%)	33 (35%)	**25 (96%)
Abdominal pain	310 (71%)	35 (36%)	**25 (96%)
Rash	127 (29%)	24 (25%)	**15 (58%)

(**p < 0.05).

**Figure 1:**
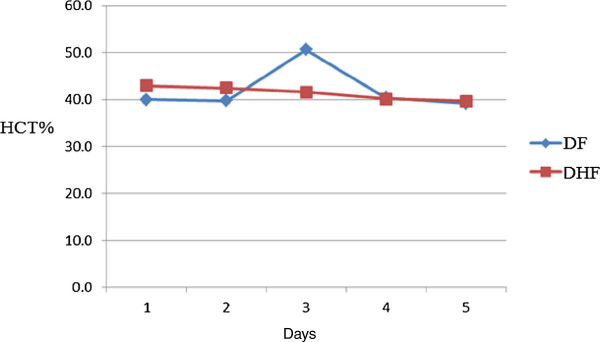
**Pattern of Hematocrit (HCT) in DF and DHF cases.** A rise in HCT was seen at 3^rd^ week in DF patients which turned to normal. However there was a consistent pattern of HCT in DHF patients. The data of DSS could not be compared as majority of them died and 05 records was not available.

**Figure 2:**
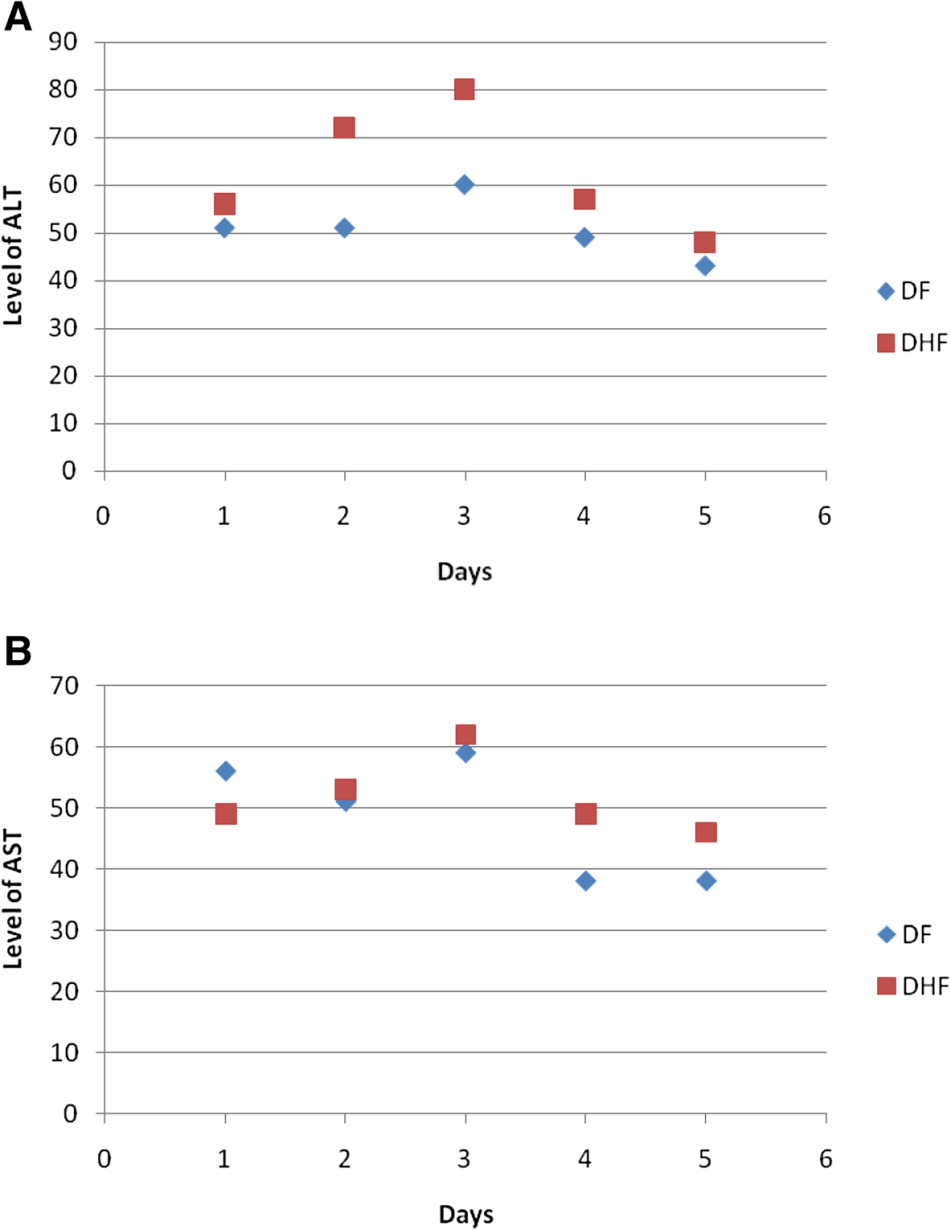
**Five days pattern of ALT (A) and AST (B) in DF and DHF cases.** The level of liver enzyme (ALT and AST) was deranged in DF and DHF cases. There was difference in level of ALT of DF cases from DHF at 3rd day of disease but this was not significant.

Five days laboratory findings showed that HCT increased at 3^rd^ day in DF cases and then returned to normal however this was constant in DHF (Figure 1). Thrombocytopenia (platelets < 100,000) was observed in 515 (93%) however 29 (5%) cases suffered from severe thrombocytopenia (platelets <10,000). ALT and AST were also de-ranged but there was no significant difference between DF and DHF (Figure 2). Records showed that platelets were transfused in 260 patients (192 DF, 48 DHF and 20 DSS) in which 19 cases had severe thrombocytopenia while in 173 cases, the count ranging from 11,000 to 40,000. About 68 (12%) patients received platelets transfusion even their counts was >40,000. The whole blood was given to 7 patients (4 DF and 3 DHF). In the treatment chart, all patients were treated in same fashion without any discrimination whether one had any other illness or not. The case record analysis showed that about 40 cases died in these hospitals. Among them 17 died of DSS, 13 from DF, 10 from DHF.

Verbal autopsy from relatives of 48 deceased was done. The average age of deceased was 43 years and 22 were unmarried. Of the 48 diseased cases, 29 (60%) were suffering from co-morbidities along with dengue infection. About 20 deceased patients had hypertension either alone or along with any other illness and majority of them suffered from DSS. Similarly diabetes and hepatitis B or C was also another major risk of developing DSS.

The clinical signs and symptoms reported by deceased attendants were: fever experienced by all patients followed by chill and vigour in 36, headache and breathlessness in 35, abdominal pain in 33, mental confusion in 35 and unconsciousness in 34 cases. Similarly chest pain was complained by 32 patients and this was sudden in onset in 12 and gradual in 9 while 11 had severe chest pain. Bleeding from nose, mouth and anus was reported before death in 30 (63%) cases. Key warning signs were severe and persistent vomiting and diarrhea about 4 days before death.

## Discussion

The present study about verbal autopsy from relatives of deceased dengue patients showed that 58% were suffering from co-morbidities. Pakistan experienced a large dengue epidemic in Lahore in 2011 resulting in thousands of deaths. Unfortunately this was sudden and health departments were not well prepared and thus there was emphasis on case management without following any local guidelines, especially for treating those with co-morbidities. There was no systematic recording, reporting and management of co-morbidities in dengue patients thus adding to the mortality rate. Several studies have reported a strong association of co-morbidities with increased complications and deaths in dengue patients [7,16-18].

Male preponderance in present study was similar to that reported in other studies [6,14,18,19]. Similarly, fever, headache, bleeding, abdominal pain and rash were common symptoms as seen previously [16,17]. Studies also show a tendency towards higher age for the deceased patients. Our study confirmed this as 62% were more than 40 years [19,20] in comparison with two studies which report mean age less than 40 years [15,21]. In about 12% cases, platelets were transfused while their counts were >40,000. Irrational platelets transfusion in dengue patients has been already reported from Pakistan [22] as well as other countries [23,24].

The patients who had previous history of any illness are more prone to develop complications. In the present study, any deceased who had hypertension either alone or along with any other illness and diabetes suffered from DSS. A study from Taiwan reported that hypertension and diabetes were risk factors for dengue hemorrhagic fever and dengue shock syndrome [25]. The warning signs in this study were severe and persistent vomiting and diarrhea which are similar to previous reports [6] in the deceased patients; fever followed by breathlessness, chills/rigor, abdominal pain, headache, stiff neck, mental confusion, unconsciousness and bleeding from mouth, nose and anus were consistent to previous reports [17,6,26,27].

There were few potential limitations in the study. Firstly, this was a retrospective analysis of available records, therefore many important information were incomplete. Secondly, data of verbal autopsies was based on the memory of deceased attendants so they might have not recalled properly.

## Conclusions

In conclusion, majority of deaths in 2011 epidemics might have been averted with better management of cases especially of those having co-morbidities.

### Future strategies

This is recommended that efforts should be done to formulate strategies for disease management especially for screening the co-morbidities and capacity building of physicians in both public and private sector hospitals to cope with such epidemics in future and to avert deaths.

## Competing interest

The authors have no competing interest.

## Authors’ contributions

MANS conceived the study, organized, interpreted the data and wrote the manuscript IR wrote the project as well as the final report and supported in data analysis and its interpretation. SB and AAS retrieved the data and interviewed the attendants of the deceased patients. All authors read and approved the final manuscript

## Supplementary Material

Additional file 1 Verbal Autopsy Questionnaire. Click here for file
