# Supplementary material for: A retrospective analysis of dengue fever case management and frequency of co-morbidities associated with deaths
Source: BMC Res Notes. 2014 Apr 1;7:205. doi: 10.1186/1756-0500-7-205 (PMC3997840; doi:10.1186/1756-0500-7-205)
Supplement: Additional file 1 — Verbal Autopsy Questionnaire. [file 1756-0500-7-205-S1.doc]

**Verbal Autopsy Questionnaire**

**Sr. No** ____________  **Date:** ________________

**A. Demographic Information of Deceased:**

Kindly confirm the name and age of patient to verify the hospital records

Name: ____________________ Age at the time of death ___________

1. Can you remember the date of expiry? Yes/ No, (if yes kindly note this)

___________________________________________________________

1. Can you please tell me the gender of deceased
   1. Male 1
   2. Female 2
2. What was his/her marital status
   1. Married 1
   2. Unmarried 2
3. What was his/her Qualification
   1. Illiterate 1
   2. Primary 2
   3. Secondary 3
   4. Above secondary 4
4. Can you please specify his/her Job/Occupation
   1. Laborer 1
   2. Service 2
   3. Business 3
   4. Unemployed 4
   5. Student 5

**B. Please tell me if deceased suffered from any of the following illnesses before dengue infection**

1. Diabetes Yes 1 No. 2 Don’t Know 8
2. High Blood pressure Yes 1 No. 2 Don’t Know 8
3. Asthma Yes 1 No. 2 Don’t Know 8
4. Epilepsy Yes 1 No. 2 Don’t Know 8
5. Malnutrition Yes 1 No. 2 Don’t Know 8
6. Cancer Yes 1 No. 2 Don’t Know 8
7. Tuberculosis Yes 1 No. 2 Don’t Know 8
8. Cardiac disease Yes 1 No. 2 Don’t Know 8
9. HIV/AIDS? Hepatitis B/C Yes 1 No. 2 Don’t Know 8
10. Did she suffer from any other medically diagnosed illness?

Yes 1 No. 2 Don’t Know 8

1. Can you specify the illness?

__________________________________________________________________

1. Any other physical co-morbidities.

________________________________________________________________

**C**. **Can you please describe the sign and symptoms of patients during hospital stay? I will ask questions one by one.**

1. Did he have a fever

Yes 1 No. 2 Don’t Know 8

1. Was the fever continuous or on and off?

Continuous 1 On and Off 2 Don’t Know 8

1. Did he/she have chills/rigor?

Yes 1 No. 2 Don’t Know 8

1. Did he/she have a cough?

Yes 1 No. 2 Don’t Know 8

1. For how long did he/she have a cough?

Days 1 Month 2 Don’t Know 8

1. Was the cough severe?

Yes 1 No. 2 Don’t Know 8

1. Was the cough productive with sputum?

Yes 1 No. 2 Don’t Know 8

1. Did he/she have cough out blood?

Yes 1 No. 2 Don’t Know 8

1. Did he/she have night sweets?

Yes 1 No. 2 Don’t Know 8

1. Did he/she have breathlessness?

Yes 1 No. 2 Don’t Know 8

1. Did he/she have chest pain?

Yes 1 No. 2 Don’t Know 8

1. For how long did she have chest pain?

Days 1 Month 2 Don’t Know 8

1. Did chest pain start suddenly or gradually?

Suddenly 1 Gradually 2 Don’t Know 8

1. When he/she had severe chest pain, how long did it last?

Less than half and hour 1 Half an hour to 24 hours 2

Longer than 24 hours 3 Don’t Know 8

1. Was the chest pain continuous or on and off?

Continuous 1 On and Off 2 Don’t Know 8

1. Did the chest pain get worse while coughing?

Yes 1 No. 2 Don’t Know 8

1. Did he/she have palpitations?

Yes 1 No. 2 Don’t Know 8

1. Did she have diarrhoea?

Yes 1 No. 2 Don’t Know 8

1. For how long did she have diarrhoea?

Yes 1 No. 2 Don’t Know 8

1. Was the diarrhoea continuous or on and off?

Yes 1 No. 2 Don’t Know 8

1. How many times did she pass stools in a day? Number 9 Don’t Know 8
2. Did he/she vomit?

Yes 1 No. 2 Don’t Know 8

1. For how long did he/she vomit?

Days 1 Months 2 Don’t Know 8

1. Did the vomit look like a coffee-colored fluid or bright red/blood red or some other?
   - 1. Coffee-Coloured Fluid 1
     2. Bright Red/Blood Red 2
     3. Other 6
     4. Don’t Know 8
2. How many times did she vomit in a day?

Number 9 Don’t Know 8

1. Did she have abdominal pain?

Yes 1 No. 2 Don’t Know 8

1. For how long did he/she have abdominal pain?

Days 1 Months 2 Don’t Know 8

1. Did he/she have abdominal distension?

Days 1 Months 2 Don’t Know 8

1. Did the distension develop rapidly within days or gradually over months?
   - 1. Rapidly within days 1
     2. Gradually over months 2
     3. Don’t know 8
2. Was there a period of a day or longer during which she did not pass any stool?

Yes 1 No. 2 Don’t Know 8

1. Did he/she have difficulty or pain while swallowing food?

Yes 1 No. 2 Don’t Know 8

1. Did he/she have headache?

Yes 1 No. 2 Don’t Know 8

1. For how long did he/she the have headache?

Days 1 Months 2 Don’t Know 8

1. Was the headache severe?

Yes 1 No. 2 Don’t Know 8

1. Did she have stiff or painful neck?

Yes 1 No. 2 Don’t Know 8

1. Did she have mental confusion?

Yes 1 No. 2 Don’t Know 8

1. For how long did he/she have mental confusion?

Days 1 Months 2 Don’t Know 8

1. Did he/she become unconscious?

Yes 1 No. 2 Don’t Know 8

1. For how long was he/she unconscious?

Days 1 Months 2 Don’t Know 8

1. Was there any change in color of urine?

Yes 1 No. 2 Don’t Know 8

1. For how long did she have the change in color of urine?

Days 1 Months 2 Don’t Know 8

1. During the final illness did he/she ever pass blood in the urine?

Yes 1 No. 2 Don’t Know 8

1. For how long did he/she pass blood in the urine?

Days 1 Months 2 Don’t Know 8

1. For how long did he/she have the skin rash?

Days 1 Don’t Know 8

1. Did he/she had rash on body?

Yes 1 No. 2 Don’t Know 8

If yes then

1. Was the rash on:
2. The face? Face 1 2 8
3. The trunk? Trunk 1 2 8
4. The arms and legs? Arms and legs 1 2 8
5. Any other place? Other place 1 2 8
6. Specify _______________
7. Did he/ She has bleeding from mouth, nose, anis?

Yes 1 No. 2 Don’t Know 8

1. 64. Did he/she have any swelling?

Yes 1 No. 2 Don’t Know 8

1. For how long did he/she have the swelling?

Days 1 Months 2 Don’t Know 8

1. Was the swelling on:
   1. The face? 1 2 8
   2. The joints? 1 2 8
   3. The Ankles? 1 2 8
   4. The whole body? 1 2 8
   5. Any other place? 1 2 8
   6. Specify _______________
2. Did she receive any treatment for the illness that led to death?

Yes 1 No. 2 Don’t Know 8

1. Can you please list the drugs she was given for the illness that led to death?

_____________________________________________________________

1. What type of treatment did she receive?

Yes No DK

1. Ors/drip treatment 1 2 8
2. Blood Transfusion 1 2 8
3. N/G feeding(Through the nose) 1 2 8
4. Other _______________________

**D. Death Certificate:**

1. Do you have a death certificate for the deceased?

Yes 1 No. 2 Don’t Know 8

1. Can I see the death certificate?

Day______ Month ______Year_______

1. Copy day, month and year of issue of death certificate?

Day______ Month ______Year_______

1. Record the cause of death from the first (top) line of the death certificate?

______________________________________________________________

1. Record the cause of death from the second line of the death certificate (if any)?

_________________________________________________________________

1. Burial place:

_________________________________________________________________

**Interviewer’s Observations**

**To be filled in after completing interview**

______________________________________________________________________________________________________________________________________________________________________________________________________________________________________________________________________________________________________________________________________________________________

Comments on specific questions:

______________________________________________________________________________________________________________________________________________________________________________________________________________________________________________________________________________________________________________________________________________________________

Any other comments:

______________________________________________________________________________________________________________________________________________________________________________________________________________________________________________________________________________________________________________________________________________________________

Supervisors observations

________________________________________________________________________________________________________________________________________________________________________________________________________________________________________________________________________________________Name of the supervisor:____________________________ Dated:_________________
